# Outcomes of bariatric surgery in patients with depression disorders

**DOI:** 10.1371/journal.pone.0221576

**Published:** 2019-08-27

**Authors:** Sergio Susmallian, Ilana Nikiforova, Shir Azoulai, Royi Barnea

**Affiliations:** 1 Department of General Surgery, Assuta Medical Center, Tel-Aviv, Israel; 2 Department of Nutrition, Assuta Medical Center, Tel-Aviv, Israel; 3 Assuta Health Services Research Institute, Assuta Medical Center, Tel-Aviv, Israel; University of Toronto, CANADA

## Abstract

**Objective:**

To determine the impact of sleeve gastrectomy in patients suffering from depression compared with those who are not in a depressive state.

**Introduction:**

Obesity is considered a global epidemic. Often patients with obesity suffer from depressive state. Depressive disorders may be both a cause and a consequence of obesity.

**Material and methods:**

The study includes 300 consecutives patients that underwent laparoscopic sleeve gastrectomy. Out of the 300 patients, 253 (84.33%) of them completed the follow up for three years.

**Results:**

Out of the 300-patients, with the average age of 41.65±11.05 years old, the ratio of males to females was 1:2. The average baseline BMI was 42.02 kg/m2. A total of 105 (35.33%) of the patients suffer from depression, which was more common in male (43%) than in female (31.5%), with statistically significant difference (p = .05). Comparing the weight loss after surgery in both groups, the mean weight loss in the depression-group was 12.0 ΔBMI and in the non-depression group was 13.03 ΔBMI, (p< .001). After three years, 94 (88.68%) patients of the depression group responded as they were optimistic and satisfied with the results of the operation, with positive changes in their lives, 8 (7.55%) did not experience change and 4 (3.77%) expressed to have worsened their depressive state.

**Conclusion:**

Laparoscopic sleeve gastrectomy is successful and leads to weight loss even in subjects who are affected by depression syndrome.

## Background

Obesity is a global problem, affecting an estimated 300 million people worldwide [1]. Obesity is considered a worldwide epidemic disease that is characterized by excess adipose tissue which contributes to numerous chronic diseases and early mortality [2,3,4].

This epidemic and devasting disease has received both national and international attention due to obesity's detrimental impact on health, the enormous economic burden it imposes and its increasing prevalence [5].

Children and adolescents also experience increasing prevalence in being overweight and becoming obese due to increased dietary intake and physical inactivity [6,7].

The genetic contribution to obesity has been elucidated involving twins, in which concordance rates for varying degrees of being overweight were twice as high among monozygotic than dizygotic male twin pairs at age 20 years [8].

Behavioral and environmental factors are largely responsible for this epidemic. Recent evidence from studies found, when comparing monozygotic, dizygotic, and virtual twins (siblings who are not biologically related, but who are the same age and were raised together from infancy) that environmental factors have a greater effect on Body Mass Index (BMI) than was appreciated previously [9,10].

The relationship between obesity and the development of comorbidities is well established in literature [11]. The main comorbidities of obesity, according to the American Heart Association/American College of Cardiology Foundation guidelines for the Management of Overweight and Obesity in Adults, include hypertension, dyslipidemia, type 2 diabetes mellitus, coronary heart disease, stroke, obstructive sleep apnea (OSA), and respiratory problems [12,13,14]. Additionally, increased body weight correlated with increased death rates for all cancers combined and for cancers at multiple specific sites [15].

A recent review concluded that the majority of studies find a prospective relationship between obesity, eating disturbances and depressive disorders (DD) [16]. However, this relationship is not unidirectional; DD may be both a cause and a consequence of obesity [17].

Additionally, people who suffer from obesity develop a higher prevalence of eating-related pathology (i.e. Anorexia, Bulimia Nervosa, and impulse regulation) as expression of their state of anxiety [18]. Stigma and discrimination are then considered chronic stressors which have a profound impact on the psychological and physical well-being of the affected individuals [19]. A variety of studies link weight stigma to direct behavioral change, such as an increase in eating behavior abnormality, psychosocial stress, and indirect effect through social relationships [20]. Therefore, there is a growing amount of research providing evidence for the complex association and interaction of weight stigma and psychopathological outcomes in individuals with obesity [21].

Due to these negative outcomes, obesity is a disease that must be treated. Conservative treatments, for severe obesity, either through drugs or life style modifications, do not provide satisfactory results in terms of weight loss, maintenance, and morbidity reduction [22]. Compared with non-surgical treatment of obesity, bariatric surgery leads to greater body weight loss and higher remission of co-morbidities [23,24,25]. The influence of bariatric surgery in patients with DD was not well known until recently.

The objective of this study is to corroborate the results of bariatric surgery in obese patients suffering from DD.

## Material and methods

The study was approved by Assuta Medical Center Institutional Review Board and for the Israel Ministry of Health, the trial was registered on the National Institutes of Health website (NCT03796325 -Unique Protocol ID: AMC 002-2018(. The consent was obtained oral, since the study is non-interventional.

The current study is a prospective, midterm follow-up study based on morbid obese patients that underwent LSGs in a single private institution, from January 2013 through December 2014. In this study, 300 consecutive patients were included, whereas LSG was their primary bariatric surgery using the same technique.

Laparoscopic sleeve gastrectomy is a type of bariatric surgery in which a gastric sleeve is constructed resecting 75% of the stomach longitudinally, using a probe to calibrate the width of the rearing stomach of 34 Fr.

The present study includes patients of both genders, from ages 18 years old and above, which underwent surgery without any surgical complication, as patients with complications are likely to aggravate their depressive situation.

All the patients were evaluated by a committee composed of bariatric nutritionists, psychologists and the bariatric surgeon, analyzing DD, jobs, eating modalities, physical activities, marital status along with weight, height, Body Mass index (BMI), diseases, and compliance for behavioral changes.

A year following the surgery, we completed the follow-up with 100% of the patients, and three years' post-surgery we lost 47 patients leading to the follow-up being completed with 253 (84.33%) patients. The reasons for the loss of follow-up patients includes: 1 patient due to pregnancy, 7 patients changed their telephone numbers, 15 patients did not respond and 24 patients refused to participate.

### Statistical analysis

All of the measured variables and derived parameters were tabulated using descriptive statistics. For the categorical variables, the summary tables provided the sample size, and absolute and relative frequencies. For the continuous variables, the summary tables provided the sample size, arithmetic mean, standard deviation, median, minimum and maximum values, and 95% confidence intervals (CI) for the means of the variables. A paired t-test was used to analyze the changes and relative changes (% change) in the weights and BMIs comparing three years and one year. A two-sample t-test was applied to analyze the differences in the weight and BMI changes from one year after the surgery via the different parameters. An ANOVA test was conducted to compare the weight loss according to different characteristics.

All of the applied tests were two-tailed, and had a p value of 0.05 or less, considered to be statistically significant. The data was analyzed using SAS® version 9.3 (SAS Institute, Cary, North Carolina) and MedCalc® statistical software version 15.3 (Digimizer, Ostend, Belgium).

## Results

### Baseline demographics

Of the 300-patient study population, 200 female patients and 100 male patients, the ratio of males to females was 1:2, and the average age was 41.65±11.05 years old. The average baseline BMI was 42.02 kg/m^2^ (Range 34.33 to 72.40 kg/m^2^).

In the cohort, 37 (12%) patients suffer from diabetes, 63 (21%) from hypertension, 151 (50.33%) from hyperlipidemia. Out of 106 (35.33%) DD patients, 81 (76.42%) suffer from mild or moderate DD and 25 (23.58%) suffer from severe DD.

The concept of DD is a state of low mood and aversion to activity, which may affect a person's thoughts, behavior, tendencies, feelings, and sense of well-being ^(26)^. Severe DD is putative as Depressed mood or a loss of interest or pleasure in daily activities for more than two weeks, mood represents a change from the person's baseline, Impaired function (social, occupational, educational) [26].

Selective Serotonin Reuptake Inhibitor are used to treat 41 (38.67%) patients in the DD group.

The DD group have an average age of 44.16 years and the group without DD have an average of 40.27 year (*p* = .140). Depression was more common in male (43%) than female (31.5%), the differences lead to a statistically significant value (*p* = .05).

The DD group had a mean BMI of 42.09 Kg/m^2^, while the non-depressive group had a mean BMI of 42.43 Kg/m^2^, giving a significant value of (*p*< .001).

Comparing the presence of Diabetes, hypertension, and hyperlipidemia in depressive and non-depressive patients, there is a higher percentage of these diseases existing in the depression-group. Hypertension is present in 34% of patients with depression, while in patients who do not suffer from depression 14.94% (*p*<.001). In the depression group 21.69% are diabetic while in the non-depression group only 7.21% are diabetic (*p*<.001). Moreover, Hyperlipidemia effects 62.26% of patients with depression and 43% in patients without depression (*p* = .001).

In the group study we found that 116 (38.66%) patients without any disease and 56 (18.66%) have multiple diseases.

Analyzing marital status before surgery, 62 (20.67) patients were single, 208 (69.33%) were married, 27 (9%) divorced, and 3 (1%) widowed.

In the depression group 16.03% of patients were single and in the non-depression group 22.95% of patients were single (*p* = .157). Patients who were married in the depression group consisted of 70.75% while in the non-depression group 67.85% (*p* = .604). Regarding divorced patients, 12.27% were in the depression- group and 7.14% in the non-depression group (*p* = .138).

Laboral activities were characterized as follows: 14 (20.67%) patients were unemployed, 221 (73.67%) patients held office jobs, 64 (21.33%) patients had an active job and 1 (0.33%) worked in heavy labor.

Physical activities were classified as follows: 224 (74.67%) patients do not perform any physical activity, 76 (25.33%) patients perform light form of activity, such as walking an average of three times a week. None of the patients in this research perform an intensive or extreme type of physical activities.

Regarding work and physical activity, there is no significant difference between the depression-group and non-depression group.

Smoking habits were designed as sporadic or non-smoking. As 3 (1%) of the patients do not smoke at all, 246 (82%) patients smoke less than 10 cigarettes a day, 25 (8.33%) patients smoke 10 to 20 cigarettes a day, and 26 (8.67%) patients smoke more than 20 a day. We found that 12.26% of the depression group and 21.13% in the non-depression group are smokers (*p* = .057).

The demographic characteristic is shown in Tables 1 and 2.

**Table 1:** Baseline demographics and clinical characteristics of study participants.

VARIABLE	MEAN	SDV	MIN	MAX
Age	41.65	11.05	18	64
Height (m)	1.66	0.08	1.55	1.74
Weight (Kg)	117.83	17.63	86	217
BMI (Kg/m2)	42.02	5.03	33	72
Gender (Female/Male)	2/1			

**Table 2:** Additional baseline characteristics.

CONDITION	NUMBER	PERCENT
No comorbidities	115	38.33%
Multiple co-morbidities	56	18.66%
Metabolic Syndrome	98	32.66%
Diabetes	36	12%
Hypertension	63	21%
Hyperlipidemia	150	50%
Depression	106	35.33%
Severe Depression	25	8.33%
Single	62	20.67%
Married	208	69.33%
Divorced	29	9.67%
Widowed	1	0.33%
Unemployed	14	4.67%
Office work	221	73.67%
Active work	64	21.33%
Heavy work	1	0.33%
No/Sporadic	246	82%
<10 daily	25	8.33%
10-20 daily	26	8.67%
>20 daily	3	1%

### Loss of body weight after three years

**Fig 1:**
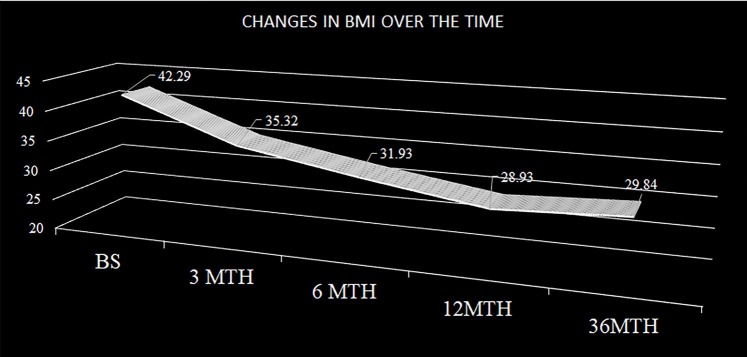
Weight loss in three years follow-up.

The mean BMI was 28.93 Kg/m2 after one year in 100% of the cohort and 29.84 Kg/m^2^ after three years postoperative in 84.33% of the patients, with a 3.14% weight gain between first and third year (Fig 1). The mean ΔBMI was 12.67 Kg/m^2^ (Range 1.6 to 42.02 Kg/m^2^).

A total of 25 (9.88%) patients did not lose enough weight to consider their bariatric operation as successful (Loss of weight less than 25% of overweight), while, 228 (90.12%) patients succeeded to loss enough weight after three years to be considered successful. The unsuccessful weight loss does not correlate with depressive patients, being that 10 of them have the condition.

Comparing the weight loss after surgery in both groups, the mean weight loss in the depression-group was 12.0 ΔBMI and in the non-depression group was 13.03 ΔBMI, giving a value of statistically significant difference (*p*< .001).

The BMI after three years with respect to different characteristics was analyzed. Women lost more weight than men but the differences were not significant (*p* = .065).

**Fig 2:**
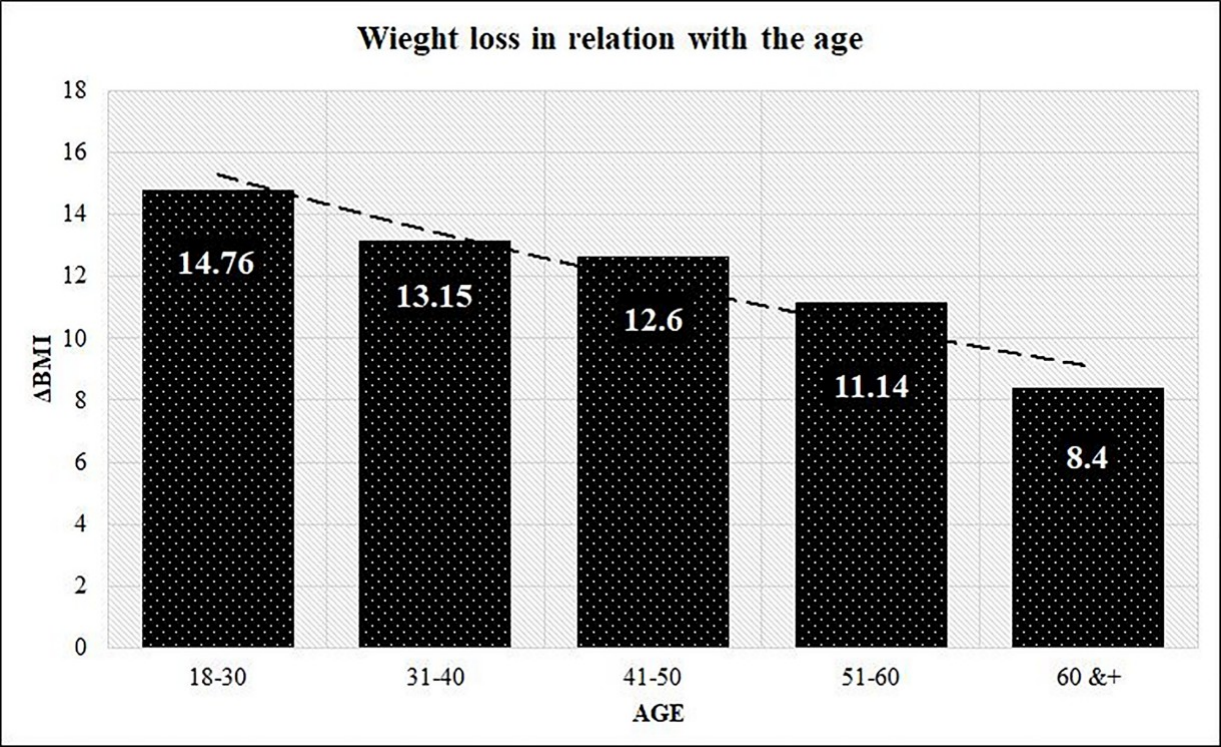
Weight loss in relation with the age.

As for age, it was found that patients under 30 years of age significantly lost more weight than patients over 30 years of age. Therefore, there was a significant correlation with decrease in weight loss and increase in age (p<.001), shown in Fig 2. Single patients lost more weight than divorced and both of those groups lost more than married patients, although the differences were not of significance (p = .123).

Engaging in physical activities, either sports or daily workouts, favored weight loss in accordance with the intensity and frequency, but the differences obtained did not show statistical significance (*p* = .427 and *p* = .479 respectively).

Dividing the patients in 4 group according to their activity, also does not affect the results post sleeve gastrectomy.

Smokers lost more weight than non–smokers, although here as well no significant differences appeared (*p* = .679).

Succeeding, weight loss was analyzed with respect to co-morbidities, depression, hyperlipidemia, hypertension, diabetes and metabolic syndrome, and no significant differences were found in terms of weight loss except in depression group. Those without depression symptoms loss more weigh that those with DD (Mean ΔBMI 13.03 and 12 respectively, P< .001) as shown in Table 3.

**Table 3:** Results of weight loss in relation with co-morbidities.

Weight loss in relation with diseases after 3 years			
Disease	N	Mean Δ BMI	P-value
Depressive	89	12.0	*p*< .001
No depressive	163	13.03	
Hyperlipidemia	128	12.29	*p* = .376
No hyperlipidemia	125	13.05	
Hypertensive	55	11.74	*p* = .276
Non-Hypertensive	199	12.92	
Diabetic	35	11.46	*p* = .260
No diabetic	219	12.86	
Metabolic Synd.	11	9.31	*p* = .097
Non-metabolic Synd.	241	12.82	

### Eating behaviors

Patient’s eating behaviors were analyzed and classified in 5 groups; volume-eaters, Binge-eaters, Sweet-eaters, Snack-eaters and patients who obtain normal eating pattern.

Preoperative eating behaviors were: binge–eater 42 (7.8%), snack–eater 142 (27.89%), sweet–eater 113 (19.89%), over-eater 249 (42.75%), normal behaviors 9 (1.67%). After three years, no significant change was found in binge-eaters (*p* = .396), 8.96%, there was an increase in snack-eater group (*p*< .001), an increase of 12.94% in sweet-eaters (*p*< .001), 17.19% healthy eating habits (*p*< .001). Sixty-five (24.8%) patients did not experience changes in their eating patterns. However, following surgery, 24.61% of the patient continue with the same EH and 125 (49.5%) patients changed from one EH to another, majority of which from the binge and sweet eaters. Weight loss measure as ΔBMI was similar in each group after three years with a mean BMI of 29.84 Kg/m2.

Analyzing the eating behaviors in both groups, DD and no DD, no differences were found (Table 4).

**Table 4:** Eating behavior in relation with depression.

Eating Behavior	Non-depression		Depression		*p*-value
	N	%	N	%	
Binge-eater	27	7.94	15	7.58	*p* = .881
Snack-eater	95	27.94	55	27.78	*p* = .968
Sweet-eater	66	19.41	41	20.71	*p* = .716
Volume-eater	148	43.53	82	41.41	*p* = .632
Normal-eater	4	1.18	5	2.53	*p* = .240

### Other changes after three years

The patients were asked to input their opinion regarding whether or not they felt the surgery was successful. Patients were asked to respond from 5 to 0, 5 being successful and 1 not being successful in general. The response for 245 (81.66%) of the patients was 5, in the depression group 87 (82.07%) patients ranked 5 points and 158 (81.44%) in the non-depression group (*p* = .893). Only 4 (1.33%) patients responded that the surgery was not successful, two patients from each group. Furthermore, the ranking of 4 was given by 27 (9%) patients, the ranking of 3 was given by 4 (1.33%) patients and the ranking of 2 was given by 3 (1%) patients. Without significative differences in both groups.

Patients were asked to classify a scale from 5 to 1 regarding whether they believed their surgery had produced the expected effects. The responders ranked as follows; 209 (69.66%) patients affirm that the results were according with the expected, 57 (19%) patients were satisfied with the results, 34 (11.33%) patients were not satisfied with the results. In the depression group 65 (61.32%) patients responded their operation as successful while in the non- depression group 144 (74.22%) patients responded positively (*p* = .021). Additionally, 286 (95.33%) patients responded that the surgery caused an improvement in the quality of life, with similar results in both groups, 11 (3.66%) patients responded with no changes in the quality of life and 3 (1%) patients thought the operation worsened their quality of life.

Changes in marital status occurred in 23 (7.66%) patients, as there were similar results in both groups.

Regarding sexual relations, 130 (43.33%) of the patients said they had noticed an improvement in their sexual relationships, 161 (53.67%) patients had no change in sexual life and 9 (3%) patients have worse sexual relations, although no differences were found in both groups.

**Fig 3:**
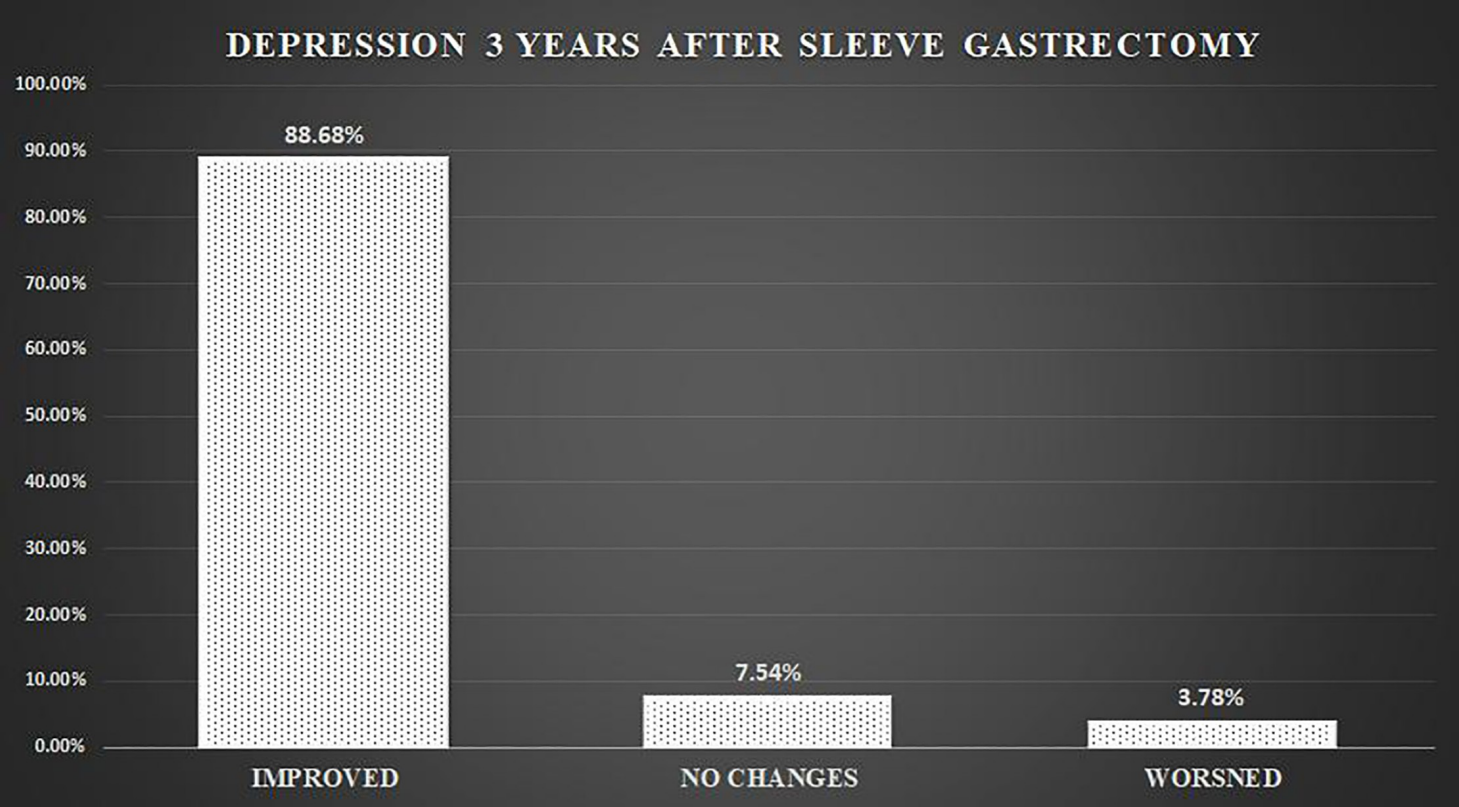
Depression state three years after laparoscopic sleeve gastrectomy.

In the group of patients with depression after three years, 94 (88.68%) of them responded feeling good, optimistic and satisfied with the results of the operation, with positive changes and effects in their lives, 8 (7.55%) do not feel change of their situation and continue to have a depressed state of mind, 4 (3.77%) expressed to have worsened their depressive situation, not being satisfied with the results and with antidepressant medication (Fig 3).

## Discussion

Obesity is a well-known cause of cardiovascular disease burden and premature death [27], but effects on psychological morbidity remains unclear [28].

Optimists expect good things to happen to them; pessimists expect bad things to happen to them.[29] Optimists and pessimists differ in how they approach problems and the success with which they cope with adversity [30]. These differences have important implications for their psychological and physical quality of life [30].

Mental health conditions are common among bariatric surgery patients—in particular, depression and binge eating disorder [31]. The relationship of obesity and bad eating habits has correlated without doubt, it appears that depression and bad eating habits have concordant and logical points.

There is inconsistent evidence regarding the association between preoperative mental health conditions and postoperative weight loss [31], leading to the importance of this study being to reveal the relationship between depression as a result of bariatric surgery.

The comparison of the group of patients without depressive features and those that do present project depressive features have no demographic differences excluding that in male depression it is more common. Herva et al. Did not find depression differences in both genders [32], another study found a small prevalence of obesity and depression in females without significative differences [33].

Significantly obese patients with co-morbidities were more likely to be depressed than those without concomitant diseases such as diabetes, hyperlipidemia and hypertension. We agree with Atlantis, in that obesity may increase the risk of depression outcomes among subgroups of individuals with specific co-morbidities [34]. Ali et Al. identified raised rates of depression in people with Type 2 diabetes [35]. While, Björntorp fault Factors activating the stress centers in humans include psychosocial and socioeconomic handicaps, depressive and anxiety traits, alcohol and smoking; several genes are associated with the cascade of events along the stress axes [36].

Sleeve gastrectomy is a restrictive type operation that produces a satisfactory weight loss in more than 90% of patients, as can be shown in the results of our study and demonstrated by other authors [37].

Greater success following bariatric surgery appears to occur in patients who are young, female, and have a high self-esteem, good mental health, a satisfactory marriage, and a high socio-economic status, have realistic expectations and undisturbed eating behaviors [38], our study corroborate similar results.

Kinzl in her study indicate a less successful outcome for obese patients with psychiatric disorders (particularly adjustment disorders, depression and/or personality disorders), compared to patients not mentally ill [39].

Eating habits is one of the determinants of obesity, but the influence of emotional traits in eating determines the way of eating. The success of bariatric surgery is based, fundamentally, on changes of eating habits and for this the improvement of the mood is essential. Study has verified the relationship that emotional eating was a mediator between depression and BMI, adjusted for age in both sexes [40].

Likewise, it has been shown that the type of food influences the state of mind. Chistensen affirm that depressed individuals have for sweet carbohydrate/fat a rich food seems to result from the enhancement in mood following consumption [41]. In another study by the same author, the results show that the mood influences the selection of food [42]. Food addiction was significantly associated with higher emotional eating, and with loss of control over consumption of foods high in fat, sugar and/or salt, but not of fruits, vegetables or grain products [43]. This may give an explanation as to why in our group of studies we found changes in food habits induced by surgery of which are drastically worse post- surgery rather than prior to surgery.

Obesity has a definite impact on quality of life, even without other comorbidities. Surgery for obesity results in significant and lasting improvements in patient-reported quality of life outcomes [44].

Body image satisfaction is associated with less depressive symptoms in all post-bariatric patients [45]. We agree with Kirkil that sleeve gastrectomy is a highly effective bariatric procedure in the manner of weight control, improvement in comorbidities and increasing of quality of life in short- and mid-term [46]. In addition, previous work that was conducted by Del Gernio demonstrated an improvement in obstructive sleep apnea syndrome following sleeve gastrectomy, implicating for another possible pathway in which bariatric surgery can improve quality of life [47].

We think that the wholesome results of sleeve gastrectomy can obtain considerable weight loss, improvement in self-esteem, decrease in anxiety, amelioration in sexual functions and general quality of life [48]. Similarly, this supports the finding in that bariatric surgery also has a positive impact on the professional sphere, providing the opportunity for unemployed patients to return to work [49].

Depressive symptoms improved significantly and rapidly after bariatric surgery, and body image dissatisfaction and self-esteem predicted change in depressive symptoms [50]. Self-esteem is not a well-known concept for bariatric surgeons and in the follow-up of the operated it is not essential, as we believe that a careful psychological support in the improvement of self-esteem can be beneficial and prevent the re-gained of weight.

On the other hand, we must not forget that depressive patients can and are capable of committing suicide after a bariatric surgery as confirmed by Neovius in his study [51].

Despite the fact that Kovacs in his study confirmed that the index of suicides is similar in operated patients than in non-operated patients [52].

This study has the limits of having analyzed the results only in sleeve gastrectomy, future studies could confirm if our results are also adapted to other types of bariatric surgery such as gastric bypass or small bowel bypass. Long-term follow-up is necessary to conclusively confirm our results.

## Conclusion

Laparoscopic sleeve Gastrectomy is an effective surgery for weight loss as the main goaleven among patients who suffers from a depressive state.

Bariatric surgery was associated with improvements in family relationships, self-esteem and produces a high index of satisfaction, mainly due to the observed weight loss. Depressive patients lost less weight than the non-depressed patient group following Sleeve Gastrectomy.
